# Development of a Near-Infrared Photoacoustic System for Selective, Fast, and Fully Automatized Detection of Isotopically Labeled Ammonia

**DOI:** 10.1021/acs.analchem.2c01191

**Published:** 2022-10-03

**Authors:** Emily Awuor Ouma, Helga Huszár, László Horváth, Gábor Szabó, Csaba Janáky, Zoltán Bozóki

**Affiliations:** † Department of Optics and Quantum Electronics, University of Szeged, Dóm tér 9, H-6720Szeged, Hungary; ‡ Department of Physical Chemistry and Materials Science, University of Szeged, Dóm tér 9, H-6720Szeged, Hungary

## Abstract

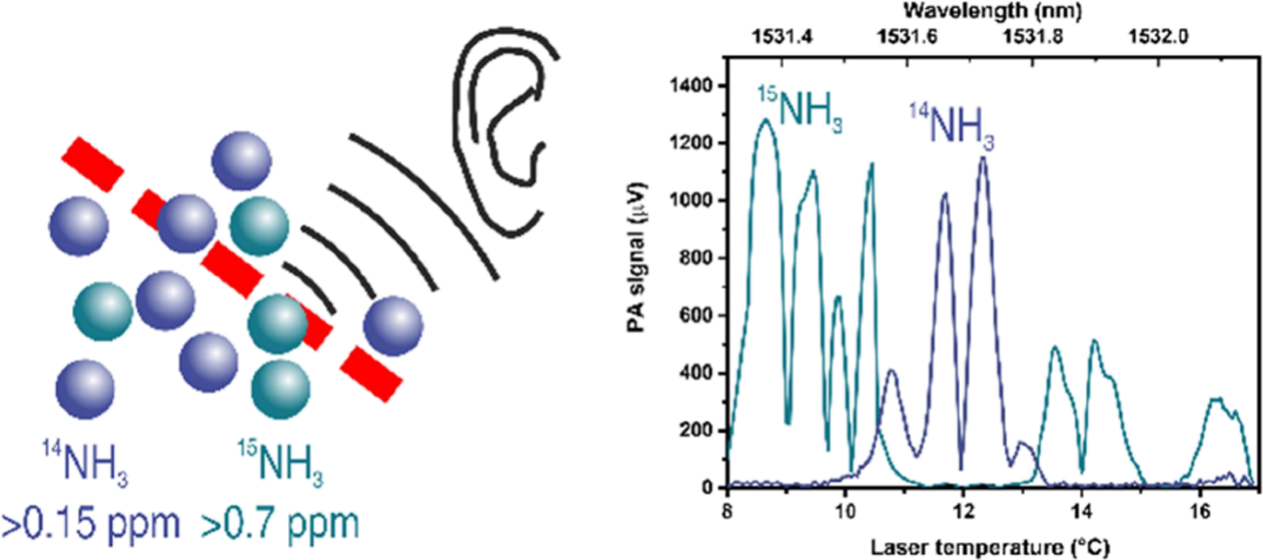

Different environmental and industrial technologies seek for fast and automatic ammonia detection systems, capable of the selective measurement of the concentration of its isotopes at sub-ppm levels, without any interference with the common contaminants. In this work, we report the quasi-simultaneous measurement of ^14^NH_3_ and ^15^NH_3_ concentrations based on a near-infrared diode laser-based photoacoustic system. Using a widely tunable external cavity diode laser, four nearby wavelengths within the range of 1531.3–1531.8 nm were optimal circumstances for sensitive detection, while avoiding interference with water vapor. Subsequently, a more robust distributed feedback diode laser was employed to tune the laser wavelength on the sub-second timescale by varying its driving current rather than using much slower temperature tuning. The detection limit of our system is 0.15 and 0.73 ppm for ^14^NH_3_ and ^15^NH_3_ (with an accuracy below 0.1%), respectively, and the response time is 3.5 s.

## 1 Introduction

Industrial ammonia (NH_3_) production through the Haber–Bosch process is a significant contributor to climate change, accounting for 1.2% of the global CO_2_ emissions. It has therefore become imperative in the scientific community to develop alternative methods for NH_3_ synthesis. Electrochemical nitrogen (N_2_) conversion to NH_3_ has become a popular research field during the past decade,^1^ as it holds the promise of substituting the energy-consuming and polluting Haber–Bosch process. Because of the infancy of this field, the NH_3_ generation rates are generally very small (often resulting in concentrations well below 1 ppm) to an extent that the detected amounts can easily originate from different contamination sources (air, human breath, N_2_ gas source, unstable N-containing compounds, etc.). In a recent article, rigorous isotopic labeling measurements were integrated in N_2_–to–NH_3_ conversion studies.^2^ Rather shockingly, it was found that the metallic catalysts, which are the most active in aqueous solutions, *did not generate any NH_3_*. This observation highlights that sensitive detection of NH_3_ is not enough, and one also needs to be able to detect ^15^NH_3,_ in the presence of ^14^NH_3_ and N_2_ gases. Such a protocol can prevent false-positive results, while also providing information on the presence of contaminants. Furthermore, to perform mechanistic studies with appropriate time resolution, we need analytical methods, which can be connected in-line and can provide (quasi)-real-time information on the product formation (i.e., separation or derivative formation-based methods are excluded). In addition, isotopologues of NH_3_ (^14^NH_3_ and ^15^NH_3_) are commonly applied in physiology, for metabolic tracing studies where they help the identification of biosynthetic pathways used by cells.^3^ Another major application is in environmental monitoring (e.g., water and air quality and exhaust gas analysis) and in industrial process control (e.g., chemical, pharmaceutical, and propulsion).

Although there is a wealth of analytical techniques and methods for NH_3_ detection, their selectivity, sensitivity, and response time with an appropriate detection limit have been an important analytical problem for decades. Starting from wet chemical methods, through physical and chemical interaction-based ones, a wide sort of spectroscopic methods (spanning through the whole electromagnetic spectrum) have been employed (Table 1). As shown in Table 1, many of the physical and chemical methods are applicable for the *bulk (nonselective)* detection of ^14^NH_3_ and ^15^NH_3_ isotopologues.

**Table 1:** Most Commonly Used Methods for Detection of Ammonia^a^

methods	principle of measurement	determination	^a^detection limit, ^b^range of linearity, ^c^sensitivity	disadvantages, interference
**physical methods for separated simultaneous measurement of ^14^NH_3_ and ^15^NH_3_**				
photoacoustic (PA) spectroscopy^4^	PA effect	detection of sound waves following light absorption in a sample	^a^10 ppb	long path length
			^c^10 ppb·m with 10 s averaging	
isotope ratio mass spectrometry^5−9^^g^^,^^10^^g^^,^^11^^f^^,^^12^^f^^,^^13^^f^^,^^14^^e^^,^^15,16^^f^^,^^17^^f^^,^^18^	partition of the mass-to-charge ratio of ions by ionization	measurement of the mass-to-charge ratio of ions	^a^0.1 ppb in the gas phase	applicable mostly in the liquid phase with extensive sample preparation
			^b^4 orders of magnitude	
nuclear magnetic resonance (NMR) method^2^^h^^,^^19^	interaction between nuclei and external magnetic field	detection of NMR signals of target nuclei excited in magnetic field	^a^10 ppb	expensive
				sensitive to contamination
				needs a large sensing volume
Fourier transform infrared spectrometry^20^^h^^,^^21^^h^^,^^22^^h^^,^^23^	multispectral absorption of materials	measurement of absorbance	^a^20–100 ppb	
			^c^<10^–15^ W·Hz^–1/2^	
				
				
**physical methods**				
PA spectroscopy^4,24−28^	PA effect	detection of sound waves following light absorption in a sample	≥^a^0.1 ppb^d^	
fluorometry^29,30^	generation of fluorescence by reagents	detection of fluorescence signal	^a^0.02 ppb in liquid phase	complex instrument for automation
differential optical absorption spectroscopy^31^	specific absorption of light by gas molecules	detection of extinction of light at a specific wavelength	<^a^1ppb	
chemical ionization mass spectrometry^32−35^	partition of ions according to the mass/charge ratio after ionization	measurement of the mass spectrum	^a^0.01–0.3 ppb in gas phase	adsorption in tubing. Problem controlling background signal
			0.04 ^c^Hz/ppt with O_2_^+^	
cavity ring-down spectrometry^36−39^	absolute extinction of laser light in a detection cavity by scattering and absorption in a specific wavelength	measurement of the decay rate	^a^10 ppb	expensive limited availability of tunable laser light at the appropriate wavelength
				
				
**chemical methods^b^**				
Nessler method^40^	transformation into colored derivatives	extinction of the light beam in ≈400 nm	^a^0.02 ppm in the liquid phase	bias by amines, chloride, and alkaline earth metals
			^b^0.02–2 ppm	
indophenol blue method^41,42^	transformation into colored derivatives	extinction of the light beam in ≈670 nm	^a^0.04 ppm in the liquid phase	Fe(III) ion
			^b^0.04–2 ppm	
ion chromatography^42,43^	separation of ammonium ions in a column	conductivity measurement	^a^0.01 ppm in the liquid phase	influence of the sample matrix
			^b^0.05–40 ppm	amines
				metal cations
annular denuder^c^^,^^42,44,45^	absorption by acids in rotating annular denuder	conductivity measurement	^a^0.01 ppb in gas phase	high cost, continuous inspection during field measurements
ammonia selective electrode^46−48^	pH change by ammonia diffusion through membrane	potentiometry	^a^0.01 ppb	ionic strength
			^b^0.01–17,000 ppm in the liquid phase	precision decreases with low NH_3_ concentration
				possible escape of gaseous ammonia

a Note: Some of the methods based on transformation of ammonia into ammonium ions for determination in the liquid phase. Most of the concentration measurements involve special sampling procedures.

b All of the methods involving chemical transformation (even NH_3_ ↔ NH_4_^+^) called chemical methods.

c It can also be used for separation of ammonia in air sample by tubular or annular denuder for subsequent ion chromatographic or spectrophotometric determination.

d Strongly depends on the light source, resonator, microphone used, and integration time.

e Equipped with a GC–MS.

f Equipped with an elemental analyzer.

g Combined with an ion chromatograph.

h Without isotope measurement.

The pool narrows drastically when an isotopic analysis is also required. Such methods are mostly based on mass spectrometry (MS), such as isotope ratio mass spectrometry for simultaneous detections of ^14^NH_3_ and ^15^NH_3_ isotopes. This method has a number of disadvantages; first of all, it involves complicated sample preparations (aqueous phase samples are analyzed), which is in many cases a source of artifact or other possible bias and error during measurements. Moreover, it is frequently combined by headspace, elemental analyzer, or ion chromatograph. The use of MS-based methods is challenging in general, because the mass difference between H_2_O and ^15^NH_3_ is very small (0.008 AMU); therefore, the suppression of the water signal becomes necessary to quantify the ^15^NH_3_ signal. This often yields to uncertainties in the detection – especially at low ammonia concentrations.^49^ Fourier transform infrared detection is based on the change in the vibrational frequencies because of the different molar masses. While this method is cheaper, easier to operate, and faster, compared to MS-based methods, it has disadvantages of requiring a considerably high amount of gas sample, low selectivity, and limited system stability.^20−23^ NMR spectroscopy is also an option, as both the ^15^NH_3_ signal and the splitting of the ^1^H resonance (because of the ^1^H and ^15^NH_3_/^14^NH_3_ interactions) can be traced. Unfortunately, both options are hindered at low concentrations and require relatively long measurement times. Overall, according to the best of our knowledge, there is no method that fulfills all the requirements listed in the first paragraph. Interestingly, most of the precedent literature specializes in the measurement of nitrogen isotope from either NH_4_^+^ ion or NH_3_ gas using various adaptations of the ammonia diffusion method.^50^ The reasons for this could be the low concentration of NH_3_ (in the sub-μg/L range), which presents a detection challenge because of the poor sensitivity of the currently used methods, mostly based on compound-specific isotope analysis (CSIA).^51,52^

Another possible measurement technique is the PA detection that in principle allows the in situ and nondestructive measurement of isotopes. PA spectroscopy is a powerful technique to measure concentrations at the low levels (from ppm to ppt ranges, depending on the available light source).^4,24−28^ In a PA detector, acoustic pressure waves recorded by a microphone are generated because of molecular absorption of modulated optical radiation in gases, liquids, and solids. In case of gases, the amplitude of the generated sound is directly proportional to the concentration of the absorbed gas component.

This work aims to develop a relatively simple, yet reliable, fully automatic, and robust system for the selective, rapid, and sensitive measurement of ammonia isotopes by using a near-infrared photoacoustic (NIR-PA) system. The PA method is efficient in determining NH_3_ concentration in general, and to the best of our knowledge, there is only one report on measuring ^14^NH_3_ and ^15^NH_3_ selectively.^4^ In this study, a procedure is used where the optical path length is of the order of 10 m through a generated plume making its application in laboratory impossible. Here, we describe a newly developed PA method for the simultaneous detection of ^14^NH_3_ and ^15^NH_3_ isotopologues, with a small and easy-to-use device.

## 2 Experimental Section

Spectral measurements, calibrations, cross-sensitivity determinations, and response time measurements were executed using the gas generation system shown in Figure S1. It has two main parts: the ammonia gas generation unit that generates various mixtures of ^14^NH_3_ and ^15^NH_3_ and the NIR-PA system, operated either by an external cavity diode laser (ECDL) or by a distributed feedback (DFB) diode laser. The gas generator unit was operated either in a mass-flow controller mixing mode or in a chemical reaction-based mode. In the first operation mode, the chemical reaction part of the system (marked by a dashed rectangle in Figure S1) is bypassed, and the calibrated mass-flow controllers are used to mix the gases from two cylinders. For the chemical reaction-based generation mode (for labeled NH_3_), a nitrogen cylinder was used to purge the gas mixture generated in the chemical reaction part of the system through the PA cell.

A longitudinal differential PA system was employed, similarly to our previous studies^27,53−56^ (see technical details in the SI). The PA spectra of the ^14^NH_3_ and ^15^NH_3_ isotopologues were recorded with gas samples either from the gas cylinders or from the chemical reaction (using pure ^15^NH_4_Cl), respectively. Measurement wavelength optimization started by recording the PA spectra of the two isotopologues and water vapor by an external cavity diode laser (ECDL, Sacher TEC 520), having an output light power of about 50 mW and its wavelength tunable between 1470 and 1590 nm. Two wavelength ranges were selected for further tests (see Discussion), from which the wavelength range of 1530.5–1533.5 nm has been chosen for further system optimization, using a telecommunication-type fiber-coupled DFB diode laser (type: FOL15DCWD-A82-19560-A, Furukawa Inc.) operating with an emitted light power of about 45 mW. This laser has been operated in a wavelength modulated mode with an unmodulated current set to be close to 300 mA, with a small amplitude sinusoidal modulation superimposed on it. Several PA spectra of the ammonia isotopes and water vapor were recorded with laser temperature tuning. For each temperature scan, the amplitude of the laser current modulation was kept fixed but changed from scan to scan. Based on the recorded spectra, an optimum laser modulation amplitude and a set of measurement wavelengths, that are least influenced by spectral interference, have been selected. Next, the measurements were accelerated by switching from temperature to current tuning. This latter operational mode was optimized in two steps: first, laser temperatures with which all the selected wavelengths are available with current tuning were screened, and then the laser temperature that provides highest possible PA signals yielding maximum sensitivity of the isotopologue concentration measurements was selected.

The concentration measurement subroutine of the operational software of the NIR-PA system was programmed in a way that after measuring at all the selected wavelengths, it converts the measured PA signals into two quantities: PA14 and PA15 (see below) characterized by high sensitivity for ^14^NH_3_ and ^15^NH_3_, respectively, while a minimal cross sensitivity against the other isotope as well as water vapor. The measurement sequence summarized in Table 2 was followed (see also a detailed description in the SI).

**Table 2:** Sequence of Procedures Applied during the Determination of the Sensitivity and Cross-Sensitivity Parameters

	measured parameter	applied gas generator	supplementary data required	the procedure for generating the required supplementary data
step 1	^14^NH_3_ sensitivity	mass-flow controller	none	none
step 2	^15^NH_3_ sensitivity	chemical reaction	actual value of *c*(^15^NH_3_)	*c*(^15^NH_3_) is determined in two steps:
				(1) determination of *c*(^14^NH_3_) from the measured value of *PA14* with the help of the ^14^NH_3_ sensitivity parameter determined in Step 1
				(2) calculation of *c*(^15^NH_3_) from *c*(^14^NH_3_) by using the mixing ratio of the isotope labeled salts
step 3	^15^NH_3_ cross sensitivity	mass-flow controller	actual value of *c*(^14^NH_3_)	*c*(^14^NH_3_) is calculated from the measured value of *PA14* with the help of the ^14^NH_3_ sensitivity parameter determined in Step 1
step 4	^14^NH_3_ cross sensitivity	chemical reaction	actual value of *c*(^15^NH_3_)	*c*(^15^NH_3_) is calculated from the measured value of *PA15* with the help of the ^15^NH_3_ sensitivity parameter determined in Step 2

## 3 Results and Discussion

**Figure 1:**
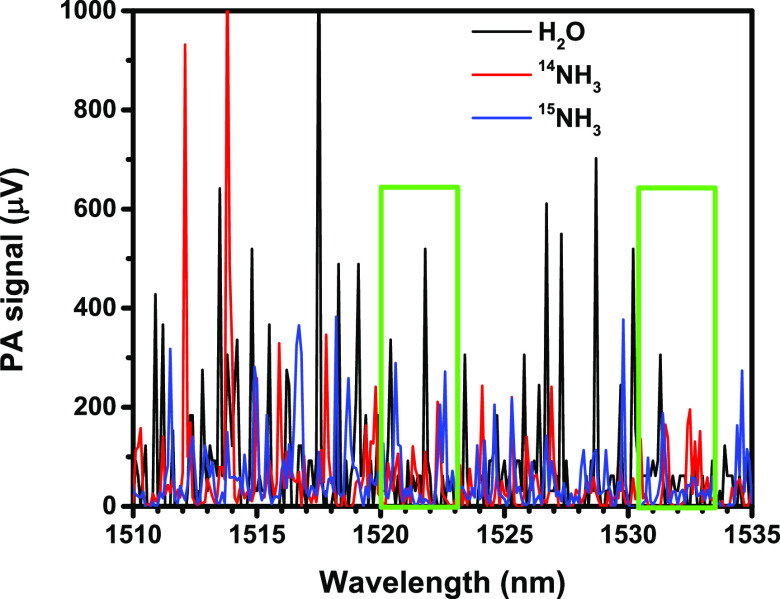
PA spectra of ^15^NH_3_ (blue line), ^14^NH_3_ (red line), and water vapor (black line) recorded by an ECDL. The two wavelength ranges marked with green rectangles were investigated in detail, by searching for optimal measurement lines.

The PA spectra recorded using an ECDL are shown in Figure 1. To select the appropriate measurement wavelengths, the analysis of the amplitude modulation generated ECDL spectra was executed by searching for less than 1 nm wide wavelength ranges, in which strong absorption lines of both isotopes can be found, while the absorption lines of water vapor are as weak as possible. On the other hand, perfect spectral separation among the absorption lines was not a selection criterion in this phase of system optimization yet, because wavelength modulation modifies the spectra considerably. Wavelengths below 1500 nm had to be excluded because of the presence of strong water vapor absorption lines. On the other hand, both the 1520–1523 nm and the 1530.5–1533.5 nm wavelength ranges met the primary selection criteria; therefore, spectral measurements with wavelength modulated DFB lasers were executed in these wavelength ranges, by attempting the minimization of spectral cross sensitivities via the optimization of the laser operational parameters. No suitable cross interference-free wavelengths in the former range were found, that is why the latter one had been selected for further optimization.

**Figure 2:**
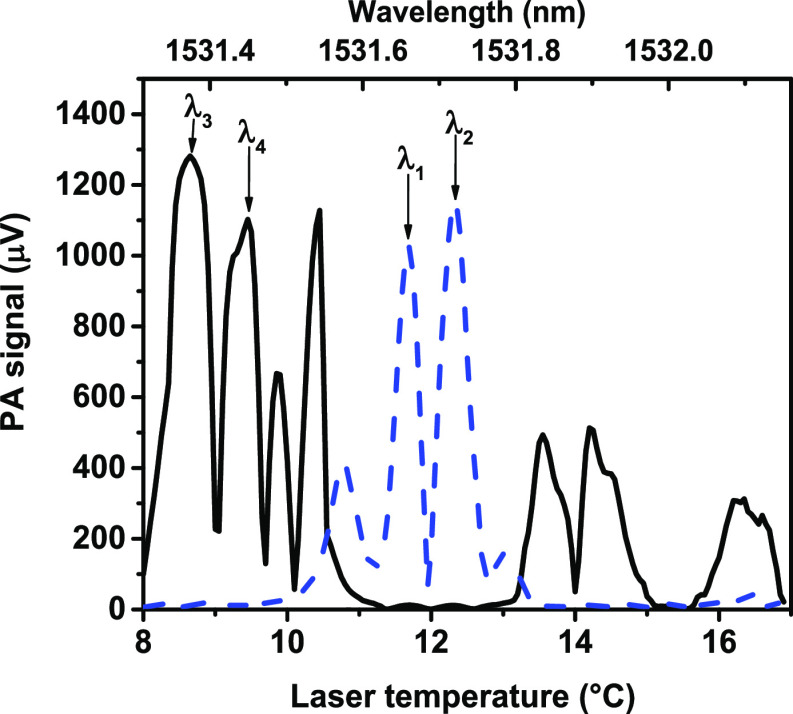
PA spectra of the ammonia isotopologues as recorded by DFB diode laser temperature tuning. Solid-black and dashed-blue lines are the recorded spectrum of ^15^NH_3_ and ^14^NH_3_, respectively. The two wavelength pairs used for selective determination of the ^14^NH_3_ and ^15^NH_3_ isotopologue concentrations are indicated as λ_1_, λ_2_ and λ_3_, λ_4_ respectively.

From the series of PA spectra of the isotopologues and water vapor, recorded by temperature tuning, the one using laser modulation amplitude of 12 mA was the least affected by spectral interferences. Indeed, these spectra contain well-separated absorption lines of both isotopically labeled compounds, while interference from water vapor absorption lines is negligible (Figure 2). The lower *x*-axis of Figure 2 corresponds to the temperature of one of the lasers, while the upper *x*-axis (i.e., laser emission wavelength) is approximated by comparing the PA spectrum of ^14^NH_3_ with data from the spectral database of PNNL.^57,58^ Based on this laser temperature to wavelength conversion, the selected measurement wavelengths are estimated to be the following: 1531.66, 1531.73, 1531.37, and 1531.45 nm, marked as λ_1_, λ_2_, λ_3_, and λ_4_, in Figure 2, respectively.

**Figure 3:**
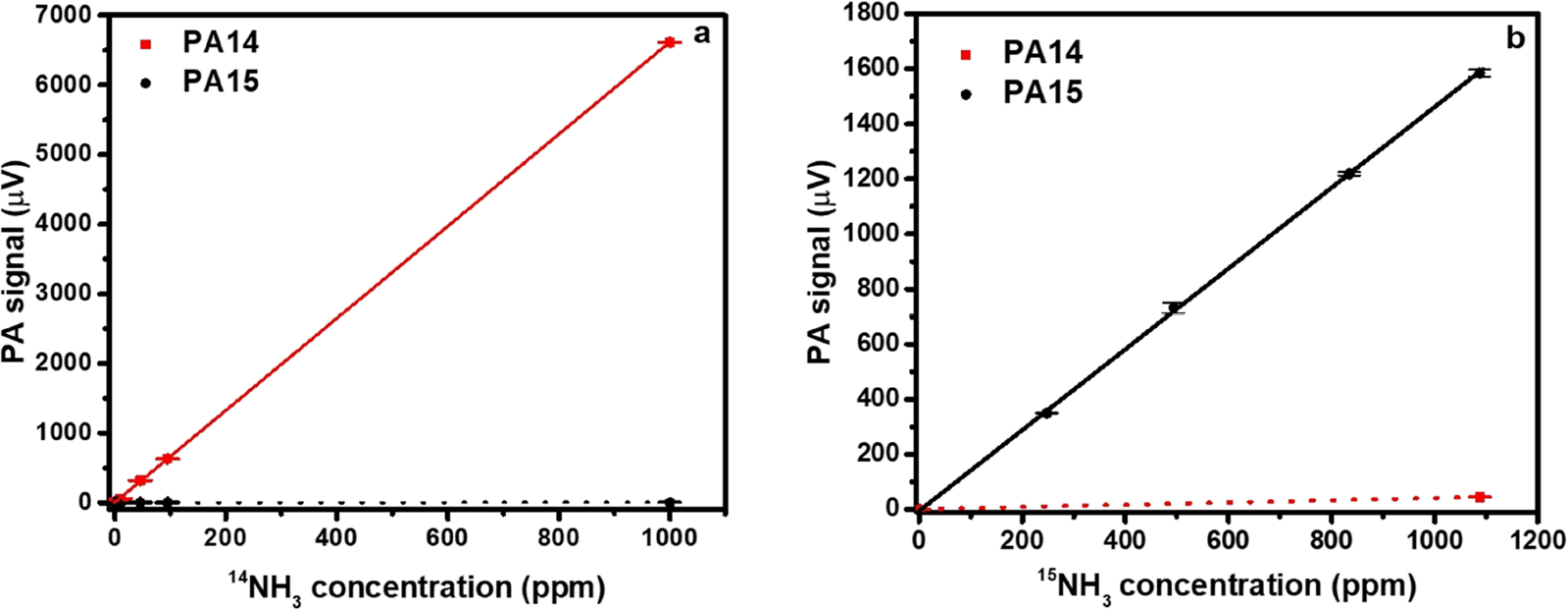
Results of the calibration and cross-sensitivity measurements of the NIR-PA system performed by the gas generation system operated either in the mass-flow controller mixing mode (a) or in the chemical reaction-based mode (b). PA14 and PA15 are the modified photoacoustic signals used to determine the concentration of ^14^NH_3_ and ^15^NH_3_, respectively. The error bars represent the standard deviation of three parallel measurements.

We note that at the beginning of the system development the possibility of using the long-wavelength end of the near infrared (e.g., the wavelength range around 2000 nm), rather than the telecommunication window, was also considered. Because of the considerably stronger absorption lines in this long-wavelength region, this is indeed very attempting, but actually there are several counterarguments as well. First, one can compare the few mW light power of a typical diode laser operating in the longer wavelength range with the ≈50 mW light power of a telecommunication diode laser. Because the generated PA signal depends on the product of the optical absorption coefficient and the light power, this product is approximately equal for the two types of lasers; that is, the disadvantage of weaker absorption lines is compensated by the much higher light power of the telecommunication diode lasers. A considerable advantage of the telecommunication-type diode lasers is their availability in a fiber-coupled construction making them very robust and facilitating their fiber coupling for increased light power. Furthermore, they have long operational lifetime (exceeding 10 years), and they are much cheaper than their long-wavelength counterparts.

Because of the narrow width (≈0.5 nm) of the wavelength range that contains all the selected measurement wavelengths, the effort to switch from temperature to current tuning was successful. The laser temperature was optimized to achieve maximum system’s sensitivity. In this optimized operation mode, the software repeatedly sets the unmodulated part of the laser to 176.3, 185, 137.2, and 147.4 mA varying the measurement wavelengths among λ_1_, λ_2_, λ_3_, and λ_4_, respectively. PA signals measured at these four wavelengths by using a modulated current of 12 mA are marked in the following as PA(λ_1_), PA(λ_2_), PA(λ_3_), and PA(λ_4_), respectively. After the completion of a measurement cycle (having all four PA signals measured), the operational software of the PA system calculates the following quantities for the quantification of *c*(^14^NH_3_) and *c*(^15^NH_3_), respectively:
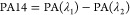

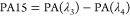


Increasing the sensitivity while simultaneously decreasing the cross sensitivity is always a primary goal of multiwavelength measurement system development. From this point of view, the definition of PA14 and PA15 as a measure of the concentration of ^14^NH_3_ and ^15^NH_3_ might be surprising at first, because Figure 2 suggests that for each isotope the subtracted signals are nearly equal, apparently resulting in reduced sensitivities. Actually this is not the case, as there is an approximately 180° degree phase difference between the subtracted PA signals; that is, PA(λ_1_) and PA(λ_2_) have opposite phases (as well as PA(λ_3_) and PA(λ_4_)), and consequently, the subtractions in the definition equations of PA14 and PA15 actually increase (almost double) these signals. This phase difference is the consequence of the applied wavelength modulation, and while it actually increases the sensitivity of the system, it is also an efficient tool for decreasing cross sensitivities. Indeed, whenever an interfering component generates roughly equal PA signals at the measurement wavelengths with the same phase, this subtraction diminishes its influence. Examples for efficiently suppressed spectral interferences include tails of absorption lines of small molecules, slowly varying absorption features generated by large molecules (or aerosol particles), and background PA signal generated by light absorption on the windows or walls of the PA cell.^59^

The result of the calibrations performed by mass-flow controllers and chemical reaction-based gas generation method is seen in Figure 3a,b. Rectangles in Figure 3a,b represent data points of PA14 vs *c*(^14^NH_3_) and PA15 vs *c*(^15^NH_3_), respectively, with the corresponding fitted calibration lines represented by solid lines. The slopes of the calibration lines give the sensitivity of 6.3 and 1.3 μV/ppm for *c*(^14^NH_3_) and *c*(^15^NH_3_) measurements, respectively. The noise of the measurements was 0.3 μV yielding 0.15 and 0.73 ppm as minimum detectable values of *c*(^14^NH_3_) and *c*(^15^NH_3_) respectively. Circles in Figure 3a,b represent the results of ^14^NH_3_ and ^15^NH_3_ cross-sensitivity determination, with the corresponding fitted calibration lines represented by dashed lines. The slopes of the fitted lines give the cross sensitivities of −1.3 × 10^–4^ and 7.4 × 10^–3^ ppm/ppm, for the measurements of *c*(^14^NH_3_) and *c*(^15^NH_3_), respectively.

**Figure 4:**
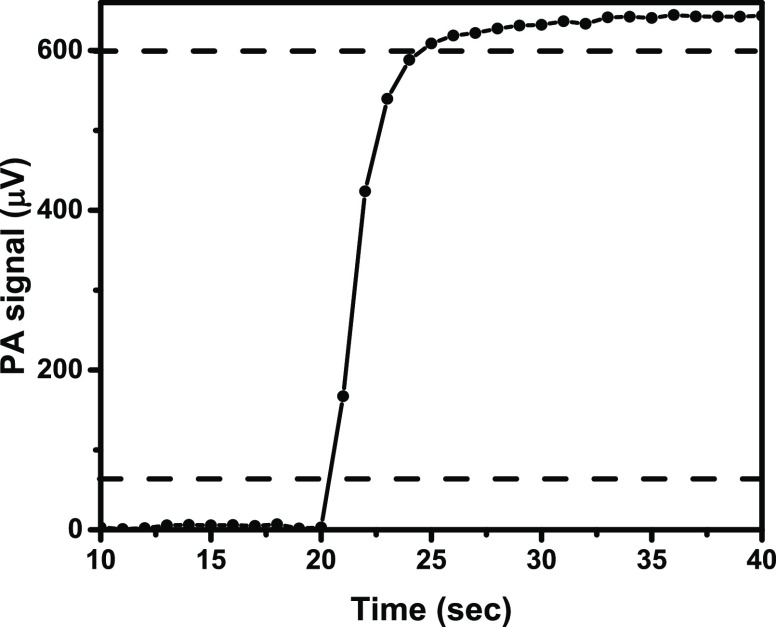
Response of the system to suddenly from 0 to 100 ppm ^14^NH_3_ concentration variation. Circles are measurement points; a line is drawn to guide the eye. The 10 and 90% of the concentration variation are indicated by horizontal dashed lines.

Finally, as shown in Figure 4, the developed NIR-PA system is capable of following sudden concentration variations with a response time of 3.5 s. Two unique properties of the system make its response time such remarkably low. First, as it is already discussed above, because of the close proximity of the selected measurement wavelengths, current tuning can be applied, and so one measurement cycle with measuring on the four selected wavelengths can be executed within less than a second. Second, the small volume (≈10 cm^3^) of the PA cell can be flushed through completely and rapidly, even at moderate gas flow rates. Roughly speaking, there is an inverse relationship between the gas flow rate and the response time, so whenever the gas production rate in the investigated process is slow, the flow rate can be reduced at the expense of the response time. At this point, it is worth comparing photoacoustics with one of their rivaling techniques: multipath optical absorption spectroscopy. It is true that the two methods have very similar analytical parameters and also that the same measurement wavelengths and current tuning method developed here can be applied with the latter technique, too. To achieve a similar response time, however, the large volume (≈1000 cm^3^) of the multipath cell has to be purged at much higher rates resulting in a considerable gas consumption. In the first approximation, the response time depends on the ratio of sample volume and the flow rate, respectively.^60^ At a high flow rate, the response time values of multipath (direct absorption) and PA methods will be comparable.^61^

## 4 Conclusions

In this work, we have developed a DFB diode laser-based PA system for the sensitive, selective, and rapid detection of isotopically labeled NH_3_. Measurement wavelengths of the isotopologues, that are among the strongest in the targeted wavelength range and lay sufficiently close to each other to facilitate the application of current tuning of the diode lasers, were selected to program the operational software of the system in a way to complete a concentration measurement cycle in less than a second. This allows fully exploiting inherently fast response of the PA detection method even in such a demanding application. Altogether, because of its robustness, high sensitivity, low cross sensitivity, and short response time the presented system is expected to find numerous practical applications ranging from electrocatalytic N_2_ conversion to biological studies.

## Supporting Information Available

Additional experimental details of the gas generation, wavelength selection, and the operation of the system (PDF)
