# Supplementary material for: Development of a Near-Infrared Photoacoustic System for Selective, Fast, and Fully Automatized Detection of Isotopically Labeled Ammonia
Source: Anal Chem. 2022 Oct 3;94(41):14118–25. doi: 10.1021/acs.analchem.2c01191 (PMC9583071; doi:10.1021/acs.analchem.2c01191)
Supplement: Supplementary file 1 — ac2c01191_si_001.pdf [file ac2c01191_si_001.pdf]

Supporting Information

Development of a Near-Infrared Photoacoustic System for Selective, Fast, and Fully Automatized Detection of Isotopically Labelled Ammonia

Emily Awuor Ouma[1], Helga Huszár[1], László Horváth[1], Gábor Szabó[1], Csaba Janáky[2,] and Zoltán Bozóki[1,]**

1. *Department of Optics and Quantum Electronics, University of Szeged, Dóm tér 9., H-6720 Szeged, Hungary*
2. *Department of Physical Chemistry and Materials Science, University of Szeged, Dóm tér 9., H-6720 Szeged, Hungary*

**Corresponding Author, E-mail: janaky@chem.u-szeged.hu*

Table of Contents

Details of the experimental processes

- Gas generation methods
- Selection and optimization of the measurement wavelengths
- Methods for determining the analytical parameters

Details of the experimental processes

The experimental setup (Figure S1) has two main parts: the ammonia gas generation unit that generates various mixtures of $^{14}NH_3$ and $^{15}NH_3$, and the NIR-PA system, operated either by an ECDL or by a DFB diode laser.

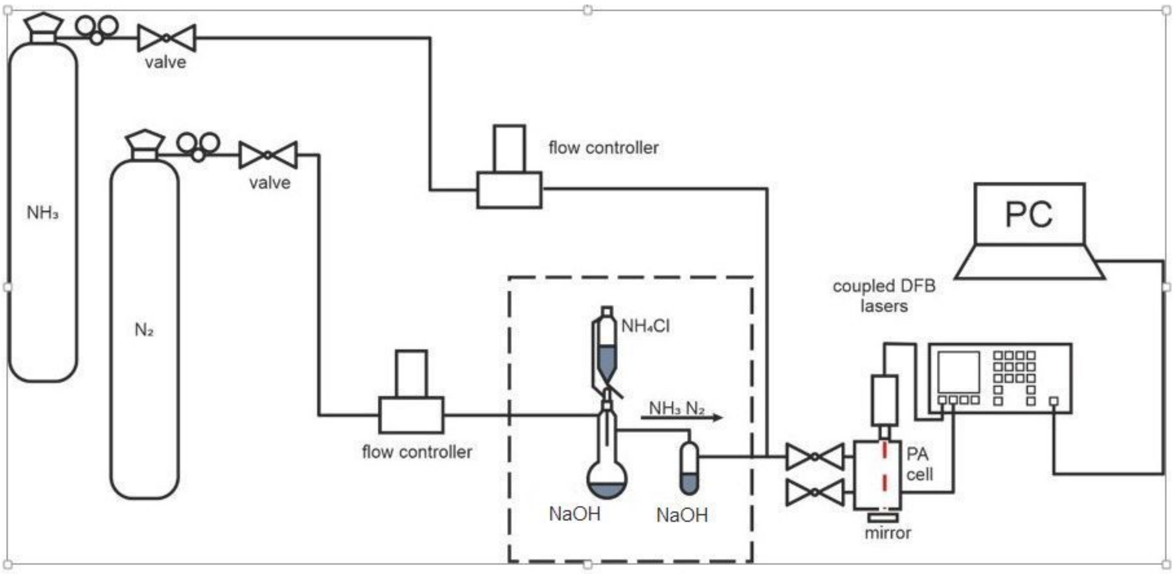

Figure S1: *Schematics of the measurement setup.*

Gas generation methods

Spectral measurements, calibrations, cross-sensitivity determinations, and response time measurements were executed using the gas generation system shown in Figure S1. It was operated either in a mass-flow controller mixing mode or in a chemical reaction-based mode. In the first operation mode, the chemical reaction part of the system (marked by a dashed rectangle in Figure S1), is bypassed and the calibrated mass flow controllers are used to mix the gases from two cylinders, one containing 1000 ppm ammonia in N_2, (in the case of response time measurement this cylinder was replaced by a one containing 100 ppm ammonia in N_2) while the other one containing pure N_2 (all cylinders have 5.0 purity and supplied by Messer). For the chemical reaction-based generation mode (for labelled NH_3) the mass flow controller of the 1000 ppm ammonia cylinder was closed and the gas from the nitrogen cylinder is used to purge the gas mixture generated in the chemical reaction part of the system through the PA cell. Gas mixture generation is based on the following chemical reaction:

$$NH_4Cl_{(aq)} + NaOH_{(s)} \rightarrow H_2O_{(aq)} + NaCl_{(s)} + NH_{3(g)} \uparrow$$

Known amounts of ammonium-^{15}N chloride (Sigma Aldrich, 98-99 atom% of ^{15}N) and ammonium-^{14}N chloride (Sigma Aldrich ≥ 99.5%) were mixed in powder form with different

mixing ratios intended to span a broad range. From each mixture, 0.14 g was dissolved in 10 ml of water, homogenized for 20–25 minutes, and then introduced into a side-tubed Erlenmeyer flask containing 0.1 g of solid NaOH in granulated form. The flask was fitted with gas inlet pipes on both sides. The nitrogen gas, used as a diluent and carrier gas, was introduced into the flask through one of its inlets at a flow rate of 150 ml/min, while the formed gas mixture enters the other tube.

Selection and optimization of the measurement wavelengths

A longitudinal differential PA system was employed, similarly to our previous studies [ref 27 in the main text], [refs 53–56 in the main text], which is characterized by a cell constant of $C \approx 20$ V·cm/W. In case the cell is illuminated with a modulated laser light having a light power of $P = 1$ mW and at the wavelength of the laser the gas within the cell has an optical absorption coefficient of $\alpha = 1$ cm^{-1}, then the microphone signal (M) generated at the laser modulation frequency, set to be equal to the first longitudinal acoustic resonance frequency of the cell, is $M \approx 20$ mV. Taking into account that the available light power (P) in a NIR-PA system can be as high as $P = 100$ mW, and the typical level of measurement noise is $N \approx 100$ nV (at 1 s signal averaging time), the noise equivalent optical absorption coefficient characteristic to our NIR-PA systems is $\alpha_{NE} \approx 5\text{x}10^{-8}$ cm^{-1}, from which the minimum detectable optical absorption coefficient (α_{MD}) can be estimated to be $\alpha_{MD} \approx 1.5 \cdot 10^{-7}$ cm^{-1} (i.e., three times of α_{NE}).

The PA spectra of the $^{14}NH_3$ and $^{15}NH_3$ isotopologues were recorded with gas samples either from the gas cylinders or from the chemical reaction (using pure $^{15}NH_4Cl$), respectively. In the former case, based on the natural abundance, the $^{15}NH_3$ fraction of ammonia is expected to be less than 0.4%. This was confirmed by the comparison of the spectra of the two isotopologues, showing that the PA spectra recorded by the gas cylinder contained no unambiguously identifiable absorption lines of $^{15}NH_3$.

Measurement wavelength optimization started by recording the PA spectra of the two isotopologues and water vapor by an external cavity diode laser (ECDL, Sacher TEC 520), having an output light power of about 50 mW and its wavelength tunable between 1470 nm and 1590 nm. Although, to the best of the authors' knowledge, there is no detailed information available on the rotational-vibrational absorption spectrum of $^{15}NH_3$ in the NIR, yet the selected ECDL was expected to provide sufficient wavelength coverage for finding the optimal measurement wavelengths. Indeed, there is a relatively strong absorption band of $^{14}NH_3$ within the wavelength range of 1480–1540 nm, and due to the small difference in their molecular weights, the absorption bands of $^{15}NH_3$ partially overlap with it. Two wavelength ranges were selected for further tests (see Discussion), from which the wavelength range of *1530.5–*

1533.5 nm has been chosen for further system optimization, using a telecommunication type fiber coupled DFB diode laser (type: FOL15DCWD-A82-19560-A, Furukawa Inc.) operating with emitted light power of about 45 mW. This laser has been operated in a wavelength modulated mode with an unmodulated current set to be close to *300 mA*, with a small amplitude sinusoidal modulation superimposed on it. Several PA spectra of the ammonia isotopes and water vapor were recorded with laser temperature tuning. For each temperature scan, the amplitude of the laser current modulation was kept fixed but changed from scan to scan. Based on the recorded spectra, an optimum laser modulation amplitude and a set of measurement wavelengths, that are least influenced by spectral interference, have been selected. Next, the measurements were accelerated by switching from temperature to current tuning. This latter operational mode was optimized in two steps: first, laser temperatures with which all the selected wavelengths are available with current tuning were screened, and then the laser temperature that provides highest possible PA signals yielding maximum sensitivity of the isotopologue concentration measurements was selected.

The sensitivity of the NIR-PA system has been further increased by adding a second DFB laser to the first one, by using a fibre coupler (FL-PBC-64-P-2-L-1-Q, Fiberlogix), and a combined power of 85 mW was reached.

Methods for determining the analytical parameters

The concentration measurement subroutine of the operational software of the NIR-PA system was programmed in a way that after measuring at all the selected wavelengths, it converts the measured PA signals into two quantities: PA14 and PA15 (see below) characterized by high sensitivity for $^{14}NH_3$ and $^{15}NH_3$, respectively, while a minimal cross sensitivity against the other isotope as well as water vapor. As usual, sensitivities were quantified as the slopes of the calibration lines fitted on the data points of PA14 and PA15 vs. $^{14}NH_3$ and $^{15}NH_3$ concentration, respectively. Minimum detectable concentration was calculated as the noise of the NIR-PA system multiplied by three and divided by the sensitivity parameter. $^{15}NH_3$ and $^{14}NH_3$ cross sensitivity is defined as the false reading of the $^{15}NH_3$ and $^{14}NH_3$ measurement caused by unit concentration of $^{14}NH_3$ and $^{15}NH_3$, respectively. Response time is defined as the time needed for the PA signal to vary between 10% and 90% of its total variation caused by a sudden concentration change.

During the calibration of the NIR-PA system, the numerical values of these analytical parameters were determined using the following measurement sequence as summarized in the Table 2 (in the main text). First, data points of PA14 signal vs. $^{14}NH_3$ concentration (marked in the following as $c(^{14}NH_3))$ were measured by using the mass flow controllers-based gas

generation method. From these data points, the $^{14}NH_3$ sensitivity was determined by a conventional calibration line fitting. Next, the $^{15}NH_3$ sensitivity was determined by using the chemical reaction-based gas generation method with various mixtures of isotopic labelled NH_4Cl. This step is complicated because of the lack of a-priori information on the concentration of $^{15}NH_3$ (marked in the following as $c(^{15}NH_3)$) in the generated gas mixtures. If the NIR-PA system is calibrated for $c(^{14}NH_3)$, the measured PA14 signal can be used to determine the actual value of $c(^{14}NH_3)$ first, from which, by knowing the mixing ratio of the isotope labelled salts, $c(^{15}NH_3)$ can also be calculated. Once $c(^{15}NH_3)$ was determined, calculation of the $^{15}NH_3$ sensitivity parameter of the NIR-PA system became straightforward by using the measured *PA15* signals. During the next step, the $^{14}NH_3$ cross sensitivity was determined by measuring the *PA15* signals of samples generated by mass flow controllers, then plotting *PA15* vs. $c(^{14}NH_3)$ and fitting a line on these data points. For this step, the $c(^{14}NH_3)$ can be calculated from the measured *PA14* using the $^{14}NH_3$ sensitivity parameter determined in Step 1. Finally, the $^{14}NH_3$ cross sensitivity is determined by measuring the *PA14* signals of the samples generated by the chemical reaction method from pure $^{15}NH_4Cl$. The value of $c(^{15}NH_3)$ required for this calculation can be determined from the *PA15* signal (measured in parallel with the *PA14* signal) using the $^{15}NH_3$ sensitivity parameter determined in Step 2.

The response time was measured by using the mass flow controllers-based gas generation system modified by adding a four-way valve to it. The gas streams from the pure nitrogen and the 100 ppm $^{14}NH_3$ containing cylinder are set to flow continuously, one of them through the PA cell, while the other through a by-pass. Both flowrates were set to 0.4 liter/minute. Switching the gas streams by the four-way valve generates the sudden concentration variation for the response time determination.
